# Width of Gene Expression Profile Drives Alternative Splicing

**DOI:** 10.1371/journal.pone.0003587

**Published:** 2008-10-31

**Authors:** Daniel Wegmann, Isabelle Dupanloup, Laurent Excoffier

**Affiliations:** Computational and Molecular Population Genetics Laboratory, Institute of Ecology and Evolution, University of Bern, Bern, Switzerland; Wellcome Trust Sanger Institute, United Kingdom

## Abstract

Alternative splicing generates an enormous amount of functional and proteomic diversity in metazoan organisms. This process is probably central to the macromolecular and cellular complexity of higher eukaryotes. While most studies have focused on the molecular mechanism triggering and controlling alternative splicing, as well as on its incidence in different species, its maintenance and evolution within populations has been little investigated. Here, we propose to address these questions by comparing the structural characteristics as well as the functional and transcriptional profiles of genes with monomorphic or polymorphic splicing, referred to as MS and PS genes, respectively. We find that MS and PS genes differ particularly in the number of tissues and cell types where they are expressed.We find a striking deficit of PS genes on the sex chromosomes, particularly on the Y chromosome where it is shown not to be due to the observed lower breadth of expression of genes on that chromosome. The development of a simple model of evolution of cis-regulated alternative splicing leads to predictions in agreement with these observations. It further predicts the conditions for the emergence and the maintenance of cis-regulated alternative splicing, which are both favored by the tissue specific expression of splicing variants. We finally propose that the width of the gene expression profile is an essential factor for the acquisition of new transcript isoforms that could later be maintained by a new form of balancing selection.

## Introduction

The sequencing and the subsequent analyses of the human genome have raised important questions about the development and maintenance of genomic complexity in our species [1], [2]. Nearly three decades ago, Walter Gilbert [3] predicted that different combinations of exons could produce multiple mRNA isoforms from a single gene, which was experimentally shown only 10 years later [4]. The report of only 20,000–25,000 protein-coding genes in our genome [5] has led to the proposal that alternative splicing acts as a major mechanism for expanding the repertoire of gene functions and for providing the large number of proteins that are necessary for complex organisms like humans [6], [7].

Alternative splicing enables a single gene to encode many different mature RNA transcripts and potentially several different protein products. Unlike promoter activity, which primarily regulates the amount of transcripts produced by a gene, alternative splicing changes the structure of transcripts and their encoded proteins. By promoting exon truncation or extension, intron retention or the inclusion/exclusion of entire exons into a mature transcript, alternative splicing potentially allows the production of very different protein isoforms with the addition or deletion of entire alternative domains [8]. During the last decade, molecular analyses demonstrated that alternative splicing determines the binding properties, intracellular localization, enzymatic activity, protein stability and post-translational modifications of a large number of proteins [9]. Essential for protein function, alternative splicing appears also to be determinant for the regulation of transcript abundance; it can indeed reduce gene expression by yielding isoforms that are degraded by non-sense mediated decay or other mechanisms [10].

In the last few years, a large number of studies have revealed the importance and ubiquity of this process in multicellular eukaryotes [11]–[13]. A complete catalogue of the types of alternative splicing occurring in humans is now available [14]–[19]. Estimations of the proportion of genes that are alternatively spliced in humans differ according to the methodology used, but they could reach 74% [20], suggesting that alternative splicing of human genes is the rule and not the exception. Other mammals exhibit a similar proportion of genes with splicing variants, but alternative isoforms are often lineage-specific, suggesting that alternative splicing is a dynamic process across evolutionary time [21]–[24]. Some studies have tried to understand how alternative splicing originated in the course of evolutionary time, through the results of mutations in DNA sequences [25]–[27] or through the evolution of splicing regulatory factors [28]. A second approach focused on the evaluation of the evolutionary trajectory of alternatively spliced genes, trying, for instance, to compare selective pressure on alternative versus constitutive exons [29], or trying to detect associations between alternative splicing and different types of evolutionary changes, such as exon creation/loss [24], exon duplication [30], [31], or Alu element-mediated exonisation [32], [33].

While the molecular mechanisms generating alternative splicing begin to be well understood, the evolutionary mechanisms allowing new splicing variants to spread and be maintained in populations have received less attention. For instance, it is still unclear if and how alternative splicing can be maintained by selection, or if it is just a transient state before the fixation of a new selectively neutral transcript form by genetic drift. If selection was involved in its maintenance, genes where splicing is polytypic (PS genes) should have different functional properties than genes where it is monotypic (MS genes). In an attempt to better understand the evolutionary forces promoting and maintaining alternative splicing, we created a database of human genes with and without splicing variants, and documented the characteristics, functional properties and expression profiles of these two types of genes. Since several studies have shown that point mutations in splice sites, splicing enhancers or silencers, may generate splicing variants for single genes [25]–[27], we developed a theoretical model of cis-regulated alternative splicing .This simple model allows us to predict the conditions for the emergence and maintenance of alternative splicing variants for genes expressed in several tissues.

## Methods

### Database of human genes with and without splicing variants

Using the gene set of the ENSEMBL database (http://www.ensembl.org/index.html, version 33.35f [34]), which corresponds to a total of 21,999 protein-coding genes, we classified these genes into two different categories: genes monotypic (referred here as MS genes) or polytypic (referred here as PS genes) for splicing. This classification was done by cross-validating the information available in the ENSEMBL dataset and the AltSplice component of the ASD-EBI database (http://www.ebi.ac.uk/asd/, release 2 [16]). The ENSEMBL annotation reports information on alternative transcripts for its genes set, but it does not directly provide data related to alternative splicing events and isoform splicing patterns for the included genes. We therefore complemented it with information taken from the AltSplice database, which is generated by an automated computational pipeline involving the comparison of EST/mRNA alignments with genomic sequences [16]. It is important to note that AltSplice uses Ensembl genes as the starting genes set for deriving splicing patterns, and it is therefore intrinsically associated with the Ensembl annotation of alternate transcripts.

It should also be noted that the AltSplice dataset only contains information relative to splicing events and not to splicing variants or transcript isoforms. Alternative splicing events lead to the occurrence of a new splicing variant. They are defined as (a) exon (or intron) isoforms where the use of alternative donor or acceptor splice sites leads to the truncation or the extension of exons (or introns); (b) intron retention where an intronic region is not spliced out; (c) cassette exons where an entire exon is seen in some transcripts but not in others; and (d) mutual exclusive exons events where the splice isoforms contain one or the other of an exon pair. Even if events and variants are clearly associated, we were not able to describe splicing variants (i.e. transcript isoforms for PS genes), because variants can differ from each other by several events.

Our resulting dataset of MS and PS genes (see Table 1) includes 9,776 MS and 4,841 PS protein-coding genes. MS genes are defined here as having a single transcript isoform documented in the ENSEMBL dataset and no documented splicing event in the AltSplice component of the ASD-EBI database. PS genes are defined as having several transcript isoforms documented in the ENSEMBL dataset, and they are associated with alternative splicing events in the AltSplice database. Since our grouping of genes only relies on the observed number of splicing patterns through ESTs, we do not distinguish between different modes of alternative splicing. Our set of PS genes therefore certainly contains genes with cis and trans-regulated alternative splicing. The number and types of splicing events for our set of PS genes is shown in Table 2, based on the information provided in the AltSplice dataset (release 2).

**Table 1:** Structure length and number of exons for human genes.

		MS genes	PS genes	Uncharacterized genes
Total number of genes		9776	4841	7382
Length of longest transcript (in kb):	average (standard deviation)	31.802 (86.333)	73.303 (117.708)	61.029 (117.053)
	[min, max]	[0.060, 2132.948]	[1.150, 1621.321]	[0.541, 2298.739]
Number of exons:	average (standard deviation)	5.481 (6.957)	18.882 (15.362)	12.595 (10.977)
	[min, max]	[1, 151]	[3, 526]	[1, 131]

**Table 2:** Number of splicing events for PS genes in humans and mice.

		PS genes (human)	PS genes (mouse)
Total number of genes		4841	3422
Number of splicing events:	mean^1^ (standard deviation)	5.554 (6.324)	3.899 (4.005)
	[min, max]	[1, 70]	[1, 59]
Cassette exons:	proportion of genes^2^	80.15%	70.11%
	mean (standard deviation)	2.928 (3.151)	2.246 (2.302)
	[min, max]	[1, 57]	[1, 34]
Exon isoforms:	proportion of genes^2^	46.56%	43.37%
	mean (standard deviation)	1.840 (1.540)	1.515 (1.067)
	[min, max]	[1, 16]	[1, 14]
Intron isoforms:	proportion of genes^2^	63.87%	64.17%
	mean (standard deviation)	2.386 (2.371)	1.889 (1.514)
	[min, max]	[1, 29]	[1, 15]
Intron retention:	proportion of genes^2^	28.86%	20.46%
	mean (standard deviation)	2.055 (1.985)	1.667 (1.283)
	[min, max]	[1, 30]	[1, 12]
Mutual exclusive exon:	proportion of genes^2^	12.04%	7.10%
	mean (standard deviation)	1.947 (2.175)	1.609 (2.439)
	[min, max]	[1, 23]	[1, 33]

1 Mean number of splicing events per gene.

2 Proportion of genes showing the corresponding type of splicing variants.

### Functional classification of genes

We used the Gene Ontology (GO) annotation [35] to characterize the function of MS and PS genes products. The GO project has developed three structured and controlled vocabularies (or ontologies) that describe gene products in terms of their 1) associated biological processes, 2) cellular components and 3) molecular functions [35]. These three ontologies and the annotation of gene products refer to the association between the genes and GO terms that correspond to functional classes. We downloaded the ontologies and the annotation of genes from the GO platform (http://www.geneontology.org/), and we used the highest level of ontology divisions as defined in the generic slim file (http://www.geneontology.org/GO_slims/) to document the functions of MS and PS genes products. This file contains a subset of the GO terms or functional classes, and gives a broad overview of the ontology content without the details of the specific terms. Additionally, we grouped several functional categories to decrease the number of GO classes for the three ontologies, as shown in Table S1. Biological processes, molecular functions and cellular components were subdivided into 9, 12 and 10 functional classes, respectively.

### Transcription analysis

Coordinates of all ESTs that mapped to the human genome were downloaded from the University of California Santa Cruz database (http://genome.ucsc.edu/, assembly May 2004). This mapping corresponds to the best BLAT [36] hit of human ESTs from GENBANK [37] to the human genome, with a nucleotide identity value falling within 0.5% of the best hit and at least 96% nucleotide identity with the target genomic sequence.

We identified all ESTs that align with a genomic sequence overlapping with an MS or a PS gene. For each mapping of an EST to a gene, we assigned an evidence for transcription for the corresponding gene. The transcription profiles of MS and PS genes were then established by linking mapped ESTs to the eVOC ontology [38] through the annotations of cDNA library sources in eVOC version 2.7. The expression profiles of the MS and PS genes were obtained for each of the four core eVOC ontologies (Anatomical System, Cell Type, Developmental Stage and Pathology). Analyses and comparisons between genes were performed by considering the first level of the eVOC hierarchy, which is shown in Table S2. Anatomical Systems, Cell Types, Developmental Stages and Pathologies were divided into 12, 31, 6 and 9 transcriptional classes, respectively. In Table S2, we only report the transcriptional classes defined in the first level of the eVOC for which expression of MS and PS genes have been found.

### Conservation of splicing patterns in mouse

We built a dataset of MS and PS genes in mouse using the same procedures as in humans. We used the mouse genes set of the ENSEMBL database (version 33.34a), which corresponds to a total of 24,741 protein-coding genes, and, as before, the AltSplice component of the ASD-EBI database (release 2 [16]). The resulting dataset for mouse (Table 2) corresponds to 13,565 MS genes, and 3,422 PS genes. The number and distribution of splicing events for mouse PS genes is shown in Table 2.

Orthologous relationships between human and mouse genes were inferred using the information provided by the ENSEMBL consortium. We first downloaded the list of human and mouse homologous genes from EnsMart version 33 [39]. Homologous genes represent the best reciprocal BLAST hits for the two species with additional pairs obtained by a combination of BLAST and location information for closely related species, such as human and mouse. We then defined as orthologous genes the pairs of human and mouse genes labeled as UBRH (Unique Best Reciprocal Hit) or RHS (Reciprocal Hit based on Synteny information) by ENSEMBL. We then classified the 16,938 orthologous gene pairs in 4 categories (MS and PS genes in the 2 species) according to our database of MS and PS genes in humans and mice. A total of 6,738 gene pairs were characterized for splicing ability in the two species.

## Results

### Chromosomal and splicing events distributions

**Figure 1:**
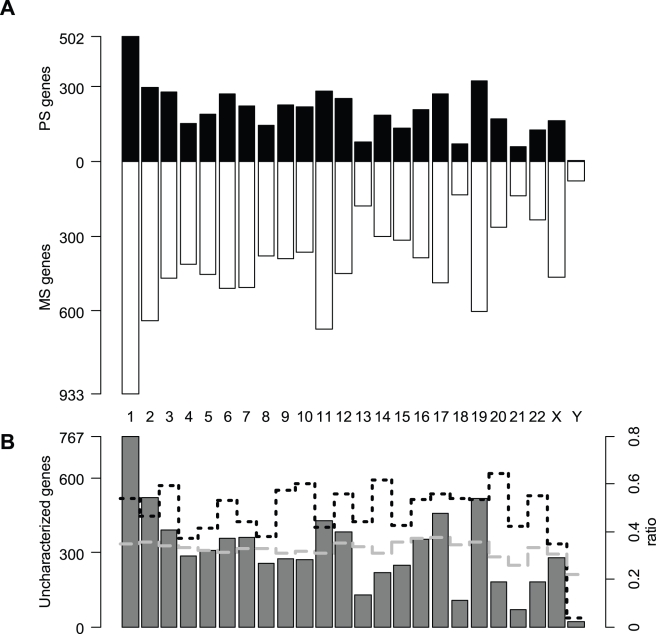
Chromosomal distribution of MS and PS genes in humans. A. Contrast between the distributions of MS (white bars) and PS genes (black bars) across chromosomes, which are significantly different (χ^2^ = 118.387, df = 23, p<0.001). B. Chromosomal distribution of genes that cannot be assigned ot the MS or PS categories. The ratio of uncharacterized genes on the total number of ENSEMBL genes (grey dashed line) is approximately constant across chromosomes. The ratio of PS genes on MS genes (black dotted line) clearly drops for the Y chromosome and to a lesser extent for the X chromosome.

The distribution of the identified 9,776 MS and 4,841 PS genes in the human genome among chromosomes is significantly different (χ^2^ = 118.4, df = 23, p<0.001, Figure 1), with a clear deficit of PS genes on the Y chromosome, and to a lesser extent on the X chromosome as well. PS genes also tend to be much longer on average than MS genes, with a larger transcript length (73.3 Kb vs. 31.8 Kb) and a larger number of exons (18.9 vs. 5.5, see Table 1). This difference is observed for all chromosomes (Figure S1A and S1AB)

PS genes show on average 5.6 splicing events but this number is highly variable (s.d. = 6.3, Table 2) Around 23% of PS genes show only 1 splicing event and the distribution of the number of splicing events per gene is skewed towards low numbers (Figure S2A). Some genes are characterized by a large number (>50) of distinct events. An example is the CD44 gene, encodeing a cell-surface glycoprotein, involved in cell-cell interactions, cell adhesion and migration, which participates in a wide variety of cellular functions including lymphocyte activation, hematopoiesis, and tumor metastasis [40]. For this gene, alternative splicing participates to the structural and functional diversity of the protein, with many variants involved in a broad range of human cancers [41]. As reported previously [42], cassette exon events outnumber the other event types for human genes (Table 2). More than 80% of PS genes indeed show at least one cassette exon and the distribution of the number of cassette exons for human genes is less skewed than the distribution of the other event types (Figure S2A).

### Gene function

**Figure 2:**
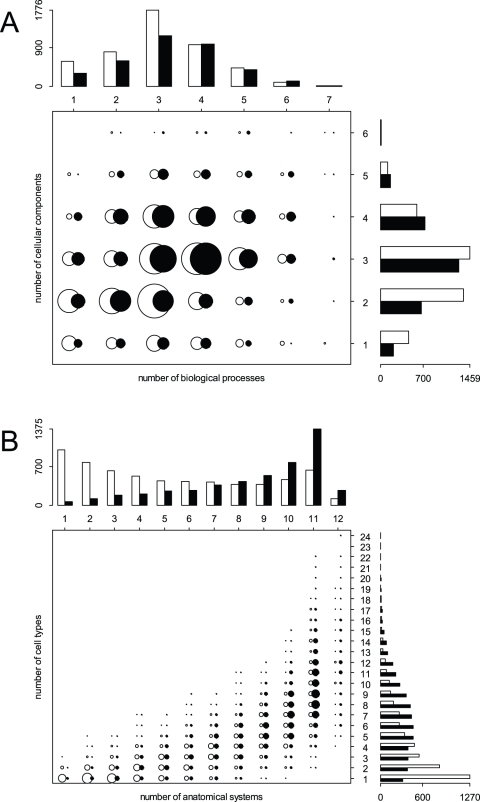
Functional classification and expression profiles of MS (white bars and circles) and PS (black bars and circles) genes in humans. A. Functional classification of genes according to the GO ontology. Note that a gene may be associated with more than one function and hence may belong to more than one functional group. The chart shows a clear and positive relationship between the number of distinct biological processes and the number of cellular components in which gene products are involved. Top (right): histogram of the number of distinct biological processes (cellular components) for MS and PS genes. The list of cellular components and biological processes is shown in Figure S4. B. Expression profiles of MS and PS genes in humans using ESTs and the eVOC ontology. The chart shows a clear and positive relationship between the number of distinct anatomical systems and the number of cell types in which the genes are expressed. Top (right): histogram of the number of distinct anatomical systems (cell types) for MS and PS genes. The cell types and the list of anatomical systems are given in Table S2. The circle areas are proportional to the number of genes. Information for MS and PS genes is shown in white and black, respectively.

We compared MS and PS genes for their Gene Ontology (GO), which is actually generally better defined for PS than for MS genes in the three GO ontologies (biological process: determined for 47.51% of MS vs. 73.35% of PS genes, molecular function: determined for 51.60% of MS vs. 81.08% of PS genes, cellular component: determined for 47.22% of MS vs. 71.12% of PS genes). We find that the distributions of the number of functional classes for MS and PS genes products are significantly different (Figure 2A and Figure S3). PS genes products are clearly involved in a larger number of distinct biological processes (χ^2^ = 995.2, df = 7, p<0.001, Figure 2A and Figure S3A), they perform a larger number of molecular functions (χ^2^ = 150.4897, df = 5, p<0.001, Figure S3B), and they are associated with or located in more cellular components than MS genes products (χ^2^ = 1042.3, df = 6, p<0.001, Figures 2A and S3C). We also observe a significant correlation between the number of functional classes for MS and PS genes products when performing a pairwise comparison of the three GO ontologies (as shown in Figure 2A and Table S3). PS genes thus show a wider functional profile than MS genes. We can also detect several differences in the distribution of genes among functional classes (Figure S4). MS genes are, for instance, enriched in biological processes related to the response to a stimulus and they are also more involved in signal transducer activity (Figure S4). In contrast, PS genes are more often involved in the regulation of biological processes, and in catalytic activities (Figure S4).

### Expression profile

We have analyzed the transcription pattern of MS and PS genes using EST databases and the eVOC ontology (see Methods). As seen in Table 3, we could assign ESTs to nearly all PS genes whereas direct evidence for transcription was only found for less than 80% of MS genes. We were thus able to characterize the expression profile in each of the four core eVOC ontologies for >91% of PS genes and for 50–60% of MS genes.

**Table 3:** Expression evidence for human genes.

	MS genes	PS genes	Uncharacterized genes
Total number of genes	9776	4841	7382
ESTs assigned	7801 (79.8%)	4834 (99.9%)	7277 (98.6%)
Anatomical system	6135 (62.%)	4815 (99.5%)	7171 (97.6%)
Cell Type	4854 (49.7%)	4654 (96.1%)	6736 (91.3%)
Development stage	5506 (56.3%)	4787 (98.9%)	7052 (95.5%)
Pathology	6073 (62.1%)	4815 (99.5%)	7164 (97.1%)

**Figure 3:**
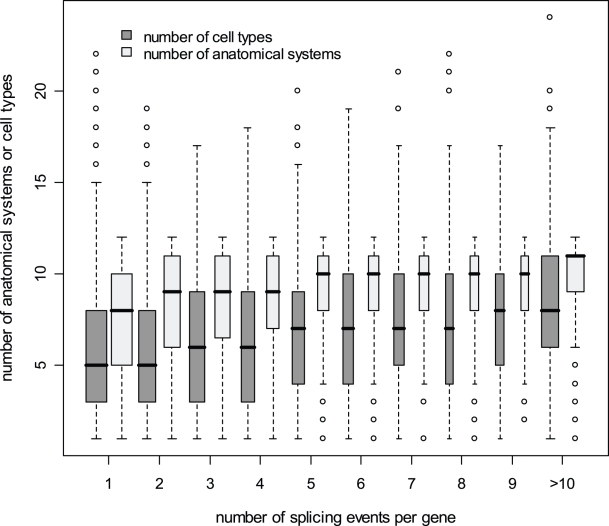
Box plot of the pattern of gene expression as a function of the number of splicing events for PS genes in humans. The number of anatomical systems is shown in light grey and the number of cell types is indicated in dark grey. The width of the boxes is proportional to the square-root of the number of genes in each group. The correlation between the number of splicing events and the width of the expression profile for PS genes is highly significant (anatomical systems: *r* = 0.224, *p*<0.001; cell types: *r* = 0.238, *p*<0.001).

The transcription profile of PS genes is found much wider than that of MS genes (Figure 2B and Figure S5). PS genes are indeed expressed in a larger number of distinct anatomical systems (χ^2^ = 2120.39, df = 11, p<0.001, Figure 2B and Figure S5A), cell types (χ^2^ = 1386.81, df = 22, p<0.001, Figure 2B and Figure S5B), development stages (χ^2^ = 1710.46, df = 5, p<0.001, Figure S5C) and pathologies (χ^2^ = 1926.54, df = 7, p<0.001, Figure S5D) than MS genes. They also present additional differences in their spatial and temporal pattern of expression, as well as in the types of pathologies in which they are involved (see Figure S6). We find an overall significant correlation between the width of expression of MS and PS genes when comparing transcription evidence across the different eVOC ontologies (see Figure 2B and Table S4). This is in line with the results of a previous study [43] showing that different genes belonging to a given biological process (GO category) have a specific breadth of expression (number of tissues where a gene is expressed), suggesting that some cellular functions are more ubiquitous than others. Interestingly, we also observe a positive and significant correlation between the number of splicing events in PS genes and the width of their expression profile (Figure 3).

### Conservation of splicing patterns in mouse

Using the same approach as in humans, we identified 13,565 MS and 3,422 PS genes in the mouse genome. The proportion of PS genes is slightly lower in mice than in humans, an observation which is consistent with earlier reports [44]. Like in humans, we observe a significant deficit of PS genes on the sex chromosomes (χ^2^ = 117.1, df = 19, p<0.001, Figure S7 A), with a striking absence of PS genes on the Y chromosome. Transcript length and number of exons are also found much smaller for MS than for PS genes (Figure S7 A and B). The distribution of the number and type of splicing events per mice gene is very similar to that obtained in humans (Table 2, Figure S2). The large majority of genes (around 92%) show less than 10 splicing events, but a few genes are characterized by a large number (>30) of distinct events. Among them the CNOT2 gene, which encodes a transcription factor which directs the formation of a diverse array of neurons and glia in the developing brain [45].

Several studies have looked at the conservation of splicing events between humans and mice, leading sometimes to contradictory results [46]–[49]. In our approach, we first identified orthologous relationships between human and mouse genes (see Methods), and then classified orthologous genes according to their splicing polymorphism in both species, based on our database of MS and PS genes in humans and mice. Orthologs in humans and mice tend to belong to the same splicing class (MS or PS, Fisher exact test of homogeneity, *p*<0.001, Table 4), showing a conservation of these categories for the genes of these two mammals. When comparing splicing events in human and mice PS genes, we note a significant correlation between the numbers of observed events in the two species (r = 0.231, p<0.001, Figure S8A), but these events differ qualitatively (Figures S8B and S8C). In other words, PS genes with many splicing events also tend to have a large number of isoforms in mice, but the splicing variants are different, which suggests a rapid turnover of transcript isoforms in mammalian history. These results are compatible with a previous study [49], which showed a highly restricted phylogenetic distribution of splicing events in mammals.

**Table 4:** Conservation of splicing ability for genes between human and mouse.

		Human dataset	
		MS genes	PS genes
Mouse dataset	MS genes	3517	1154
	PS genes	606	1461

Orthologs in humans and mice tend to belong to the same splicing class (Fisher exact test of homogeneity, p<0.001).

### Model of cis-regulated alternative splicing

While the proportion of genes among our set of PS genes with cis-regulated alternative splicing is unknown, we introduce here a simple model for the evolution of cis-regulated alternative splicing. Our goal is to describe the fate of splicing variants under the action of selection once they have appeared in a large population, and to monitor the fitness of the population as a function of allele frequencies, splicing variant fitness and expression levels. We shall not consider here the molecular mechanisms that generate new splicing variants for individual genes, since these processes have received much attention elsewhere [25]–[27]. Our evolutionary model will however allow us to state the conditions for the maintenance of existing splicing variants in populations, and therefore the maintenance of alternative splicing per se.

We consider here a simple model of cis-regulated alternative splicing, where a given gene has two alleles coding for two distinct splicing variants labeled *P*_1_ and *P*_2_. Our model thus strictly accounts for a cis regulation of alternative splicing since allelic (nucleotide) differences are supposed to fully specify the two different transcript isoforms (see e.g. [25]–[27] for examples of cis-determination of splicing variants). We further assume that the two splicing variants show different expression levels and have different associated fitness in two arbitrary tissues *T*_1_ and *T*_2_. Relative expression levels in these tissues are noted as *α_ij_*, and splicing variant specific fitness as *ω_ij_*, such that, for instance, *α*_12_ is the expression level of protein 2 in tissue 1, and *ω*_21_ is the fitness of protein 1 in tissue 2. Note that for simplicity, we shall assume the two variants are the only expressed transcripts, such that *α_i_*_1_+*α_i_*_2_ = 1 for each tissue. The tissues *T*_1_ and *T*_2_ are also supposed to contribute by a proportion *β* and 1−*β*, respectively, to the global fitness of an individual.

Under this model, the fitness of the homozygotes *P*_1_ *P*_1_ and *P*_2_ *P*_2_ are simply given by *ω_P_* = *βω*_11_+(1−*β*)*ω*_21_ and *ω_Q_* = *βω*_12_+(1−*β*)*ω*_22_, respectively, while the fitness of the heterozygotes is *ω_H_* = *β*[*α*_11_ *ω*_11_+(1−*α*_11_)*ω*_12_]+(1−*β*)[(1−*α*_22_)*ω*_21_+*α*_22_ *ω*_22_]. With this formulation, the evolution of allelic frequencies, and thus the evolution of alternative splicing, in (infinitely) large populations can be studied by classical equations of deterministic selection. For instance the fitness of the population at a given point in time is obtained classically as *ω̅* = *p* ^2^ *ω_P_*+2*p*(1−*p*)*ω_H_*+(1−*p*)^2^ *ω_Q_* where *p* is the frequency of the *P*_1_ allele, and an equilibrium frequency for *P*_1_ can be derived by solving *dω̅*/*dp* = 0, leading classically to *p_e_* = (*ω_H_*−*ω_Q_*)/(2*ω_H_*−*ω_P_*−*ω_Q_*) or
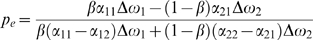
(1)where Δ*ω*_1_ = *ω*_11_−*ω*_12_ and Δ*ω*_2_ = *ω*_21_−*ω*_22_, which shows that equilibrium frequency only depends on the difference in fitness between the two splicing variants in both tissues, and not on absolute fitness values.

### Fitness landscape

**Figure 4:**
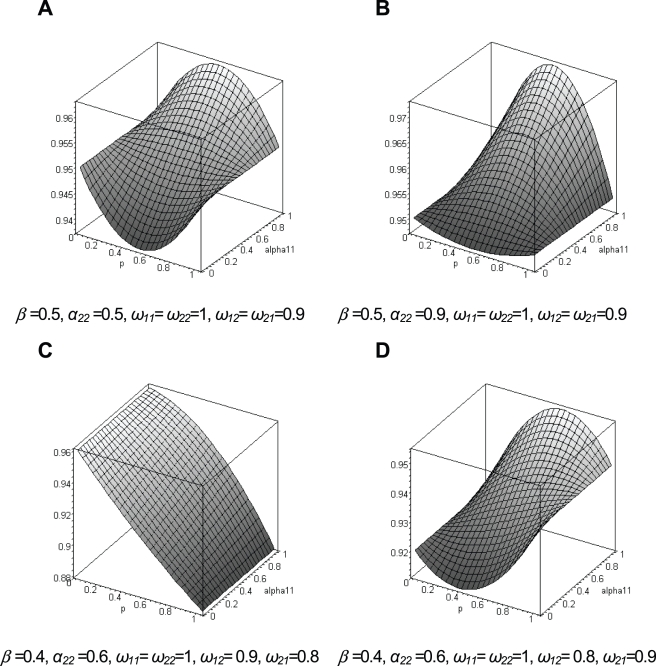
Population fitness landscape (z-axis) as a function of the frequency (*p*) of the alternative splicing variant *P*_1_, and its specificity for tissue *T*_1_ (*α*_11_). See text for the definition of the other parameters.

An examination of the fitness landscape in a few particular cases is instructive, as it can reveal if selection will promote the fixation of a new variant, or if alternative splicing can be maintained by (balancing) selection, and, if yes, what are the conditions favoring this maintenance. In Figure 4, we show the fitness of the population as a function of the frequency of the splicing variant *P*_1_, and its specificity *α*_11_ for tissue *T*_1_. In Figure 4A, we report a case where the two variants are expressed at a similar level in tissue *T*_2_ (α_22_ = 0.5), and where the two splicing variants have maximal fitness in different tissues. Here the fitness of the population is maximized when *α_11_* = 1, which corresponds to the case where *P*_1_ is the only variant expressed in tissue *T*_1_. Interestingly, selection maintains a balanced polymorphism of the two splicing variants at *p*_e_ = 0.5 for large values of *α_11_*, while disruptive selection prevails for low values of *α_11_*. In Figure 4B, the conditions are identical except that the variant *P*_2_ is much more expressed than *P*_1_ in tissue *T*_2_ (*α_22_* = 0.9), which leads to a larger portion of the landscape where balancing selection can occur. For complex cases, with unequal contribution of different tissues to the individual fitness, tissue specificity for *P*_2_ in *T*_2_ and asymmetric relative fitness components, the fitness landscape can be extremely different depending on the specific values of the parameters. Figure 4C represents a case of directional selection, preventing the form *P*_1_ to increase in frequency, mainly because *ω_12_*>*ω_21_*. Under the same conditions as in Figure 4C, but this time with *ω_12_*<*ω_21_*, the landscape becomes more complex, and *P*_1_ can increase in frequency and go to fixation or reach a stable equilibrium depending on the value of *α_11_* (Figure 4D).

### Evolution of tissue specificity

The population fitness *ω̅* can be expressed as a linear combination of the tissue specificities *α_11_* and *α_22_* as *ω̅* = *c*_1_ *α*_11_+*c*_2_ *α*_22_+*c*_3_, where *c*_1_ = 2*p*(1−*p*)*β*(*ω*_11_−*ω*_12_), *c*_2_ = 2*p*(1−*p*)(1−*β*)(*ω*_22_−*ω*_21_), and *c*_3_ = *β*(*ω*_12_+*p* ^2^(*ω*_11_−*ω*_12_))+(1−*β*)(*ω*_21_+(1−*p*)^2^(*ω*_22_−*ω*_12_)). It shows that the population fitness is maximized when the two splicing variants are not expressed in the same tissue (when *α_11_* = 1 and *α_22_* = 1), irrespective of the other parameters and splicing variant frequencies. It suggests that if splicing variant expression is free to evolve in different tissues, selection should promote tissue specific expression.

### Fate a new alternative splicing variant

The population fitness landscape will condition whether a new alternative splicing variant will be able to increase in frequency in the population. The fate of a new variant under the influence of selection alone can thus be predicted by evaluating the slope of the fitness landscape at *p* = 0. We therefore need to evaluate 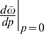, which has the simple solution 2*βα*_11_Δ*ω*_1_−2(1−*β*)*α*_21_Δ*ω*_2_.

*P*_1_ can therefore invade the population if *βα*_11_Δ*ω*_1_>(1−*β*)*α*_21_Δ*ω*_2_. Favorable conditions for this invasion are when the new variant has a high specificity for an important tissue and that its fitness advantage over the other variant in that tissue is larger than its relative fitness disadvantage in another tissue.

### Maintenance of alternative splicing

**Figure 5:**
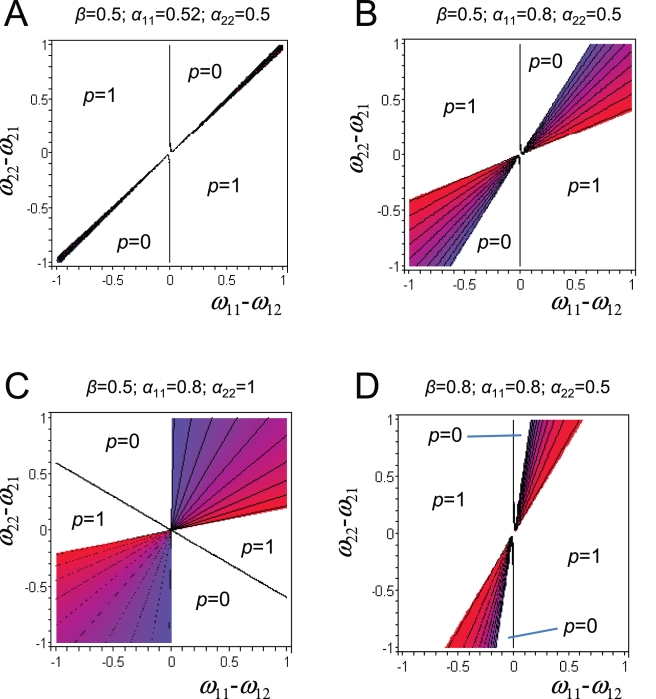
Effect of tissue specificity on the maintenance of alternative slicing. Colored zones correspond to parameter areas where alternative splicing can be maintained by balancing selection. Equilibrium frequency (*p*) can vary between 0 (blue) and 1 (red).

Another question of interest is the maintenance of a balanced polymorphism for alternative splicing in the population. As explained above, it should depend on the respective fitness of the variants in different tissues and on tissue specificity. From equation (1), we find that the conditions under which alternative splicing can be maintained by selection are
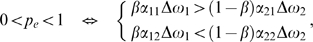
(2)In Figure 5, we report the portions of the relative fitness space where alternative splicing can persist for different levels of tissue specificity. We see that there is a much wider range of conditions leading to a balancing polymorphism when there is strong tissue specificity for alternative splicing variants, than when splicing variants are expressed at a similar level in all tissues. Indeed, in Figure 5A, when there is only a slight overexpression of *P*_1_ over *P*_2_ in *T*_1_, conditions for balancing selection are very limited and in most of the cases one alternative splicing variant will fix in the population. On the other hand, when *P*_2_ is the only variant expressed in *T*_2_, and almost not expressed in *T*_1_ (Figure 5C), almost all conditions where Δ*ω*_1_ and Δ*ω*_2_ have the same sign lead to a balanced polymorphism. The comparison of Figure 5B and Figure 5D shows that balancing selection can more easily operate when tissues are equally important for the individual fitness (*β*≈0.5). As seen previously, if tissue specificity was free to evolve, one would predict that splicing variants should become very tissue specific, which would further increase the chance for the establishment of a balanced polymorphism.

### Transient alternative splicing at haploid loci

For genes with cis-regulated alternative splicing that are present on a haploid chromosome (e.g. Y chromosome in humans), the situation is simpler and different than for diploid loci. Individuals *P*_1_ and *P*_2_ have fitness *ω*_1_ = *βω*_11_+(1−*β*)*ω*_21_ and *ω*_2_ = *βω*_12_+(1−*β*)*ω*_22_, respectively, and *P*_1_ will simply fix in the population if *ω*_1_>*ω*_2_. An equilibrium exists if *ω*_1_ = *ω*_2_, i.e. when *β*Δ*ω*_1_ = (1−*β*)Δ*ω*_2_, but this equilibrium is unstable, and one or the other splicing variant should fix in the population. It follows that unlike in the diploid case, cis-regulated alternative splicing cannot be maintained by selection alone, and that any observed polymorphism for alternative splicing should only be transient.

## Discussion

Our comparison of human genes with (PS genes) and without (MS genes) evidence for alternative splicing shows that PS genes are generally longer and contain more exons than MS genes (Figure 1), in line with the fact that longer genes have more exons and thus could produce more alternative variants. PS genes are also involved in more cellular functions and expressed in more tissues (Figure 2). Another striking difference is the clear deficit (compared to autosomes) of PS genes on the Y chromosome, and to a lesser extent on the X chromosome. A similar analysis on the mouse genome reveals very comparable patterns, including a total absence of PS genes on the mouse Y chromosome. This results extends previous indirect evidence of different levels of alternative splicing on mammalian chromosomes, where it was postulated that negative selection pressure against premature protein truncation was reduced by alternative splicing and nonsense mediated decay on diploid chromosomes but not on the X chromosome [50].

The observations that the splicing status of orthologous genes is conserved between humans and mice (Table 1, i.e. PS genes in humans tend to also be PS genes in mice), but that there is no real conservation of the splicing events (Figure S8), implies that orthologous PS genes have different sets of splicing variants in humans and mice. The conservation of the ability to have splicing variants rather than particular splicing variants in different mammal species also suggests a relatively rapid turnover of splicing variants within species. Since orthologous genes are likely to be involved in similar functions in mice and humans, we also expect their width of expression to be similar. Orthologous genes should therefore show similar opportunities for tissue specificity. According to our model of cis-regulated alternative splicing, splicing variants would be kept in the population due to balancing selection acting on tissue-specific variants, which would explain the conservation of the splicing status between human and mice. As different variants are competing, changes in form or functions of tissues between these two species are likely to bring along changes in splicing events, explaining the high turnover of splicing events observed. Note that a strict comparison of the alternative splicing variants themselves is impossible with current EST databases. This would indeed require information on complete mature mRNA or protein sequences, which seems difficult with extant techniques.

### Database integrity

Our results showing drastic constitutive difference between PS and MS genes are strongly dependent on the quality of our database on MS and PS genes in humans and mice. By cross-validating information on alternative splicing between the ENSEMBL and the ASD databases, we discarded a large proportion of humans and mice genes (>30%) from our dataset, because of incongruent annotation in the two databases. Discarded genes seem to be enriched in PS genes, since our retained data set contains only 33% (and 20% respectively) of PS genes in humans (and mice), which is much below standard estimates of this proportion (>50%) in humans and mice [20], [51]. However, if we pool the uncharacterized and the PS genes together (Figure 1, Table 1, Figures S1, S3, S5 and S7), we still detect significant differences in the chromosomal distribution, structural characteristics, functional and transcriptional profiles of MS and PS genes (results not shown).

Since the probability to detect mRNA isoformes depends on the number of ESTs sequenced for a given gene [44], the much lower number of ESTs sequenced in mice than in humans (4.3 vs 7.8 million ESTs) might at least partly explain the observed smaller proportion of PS genes in mice. We indeed find a much lower average number of ESTs mapped to MS genes than PS genes in humans (72.56 vs 296.39, t-test, p<2.2×10^−16^). Hence, the observed differences in expression patterns between MS and PS genes might therefore be artificial, as genes with a larger and wider expression pattern are more likely to be sampled by ESTs. Additionally, given that the splicing machinery is not 100% efficient, all genes are expected to produce some aberrant mRNA isoformes [52]. Hence, the probability to detect splicing variants is larger for genes bearing a larger number of exons. For instance, the average number of exons per gene on the Y chromosome is 4.42, while the genome wide average is 7.49. Further, the missing conservation of splicing variants between humans and mice is consistent with the hypothesis that many mRNA isoformes result from aberrant splicing events.

If the number of sequenced ESTs per gene and the number of exons indeed explained differences between MS and PS genes, we would expect to see a relationship between these two factors and the number of known splicing events per PS gene. By performing an ANOVA, we indeed find a highly significant relationship between the number of known splicing events and the number of sampled ESTs (p = 5.2×10^−10^), the number of exons (p = 7.4×10^−7^) and the interaction of both (p = 1.1×10^−4^). However, these factors only explain very little of the total variance in the number of splicing events among genes (*R* ^2^ = 0.061), and we are thus confident that our results are not due to a bias in the constitution of our database. Additionally, it is hard to judge whether mRNA isoforms are found more often in genes with higher expression levels or whether PS genes show a higher expression level *per se*, since genes with a wider expression pattern should also have more opportunities for tissue-specific adaptation. Note that a similar type of argument can be made for the number of exons.

We have shown that PS genes are involved in more cellular functions and expressed in more tissues than MS genes (Figure 2), which could result from the fact that alternative splicing variants of widely expressed genes could be easier to detect (see above). On the other hand, Lercher et al. [53] have shown that housekeeping gene (defined in [53] as genes that are expressed in more than eight tissues) are not randomly distributed in the genome. In particular, they seem extremely rare on the Y chromosome and under-represented on the X chromosome. This might potentially explain the observed deficit of PS genes on the sex chromosomes. To test this hypothesis, we have tried to obtain the null distribution of the number of PS genes one would observe on X and Y chromosomes by keeping the distribution of the breadth of expression constant on these two sex chromosomes. This has been done with the following procedure: 1) 100,000 random X and Y chromosomes were generated *in silico* by replacing each gene on these chromosomes with a gene randomly chosen from the whole genome having the same breadth of expression (as measured by the number of anatomical systems or cell types they are expressed in); 2) the number of PS and MS genes on the artificially created chromosomes were each time counted to get their empirical null distribution: 3) The P-value of the observed numbers were then obtained from this null distribution. This procedure reveals that the number of PS genes on the X chromosome is not significantly small (p = 0.25 when controlled for anatomical systems, and p = 0.32 when controlled for cell types). On the other hand, we find a slightly significant deficit of PS genes on the Y chromosome (p = 0.015 when controlled for anatomical systems, and p = 0.05 when controlled for cell types). This suggests that the genes located on the Y chromosome show less alternative spliced forms than those of the rest of the genome irrespective of their breadth of expression. This is in keeping with the proposed model of cis-regulated alternative splicing we have developed, where alternative splicing is actively maintained by balancing selection (see below).

### Model of cis-regulated alternative splicing

In our theoretical model of cis-regulated alternative splicing, PS genes are simply genes with more than a single splicing variant, and MS genes have a single splicing variant because newly created splicing variants could not increase in frequency or because a new variant had fixed in a population. It is important to understand that observed PS genes can therefore either be transiently so (between two fixation events due to directional selection) or be actively maintained polymorphic by balancing selection. Our model explains the strong deficit of PS genes on the Y chromosome by the inability of balancing selection to operate on this haploid chromosome. Interestingly, this observation also suggests that trans-regulation of alternative splicing, even though certainly possible [54], plays a minor role, since one would expect that it would have the same effect on autosomes and sex-linked markers. The prediction of our theoretical model that alternative splicing is favored by a strong tissue specificity in splicing variant expression fits well with the observation that PS genes are expressed in more tissues than MS genes (Figure 2B). There is actually some evidence that splicing variants have some tissue specificity or show a specialized function [55]. While the exact factors conditioning tissue specific gene expression are not well defined, it seems logical to consider that the chance for a new variant to be overly expressed in a random tissue would increase with the number of tissues where it is expressed. Therefore, the conditions for the occurrence of balancing selection would increase with the width of tissue expression taken as a proxy of tissue possible specificity (Figure 4). Interestingly, our model also shows that tissue specific expression is favored by selection, since the fitness of the population should increase with tissue specificity. However, the same variants should remain for a sufficiently long time in the population for tissue specificity to evolve, which may not be the case due to the apparent rapid turnover in splicing variants within species.

While our theoretical model of the evolution of alternative splicing is purely deterministic, genetic drift could also be involved in the segregation of splicing variants in populations, like any other molecular marker [56]. However, splicing variant frequencies should evolve under selection if the difference in variant fitness times the population effective size is larger than 1 [57] (i.e. if *N_e_*Δ*ω*_1_>1 and *N_e_*Δ*ω*_2_>1). A simple correlation between gene length and the probability of emergence of new alternative splicing variants could explain some of our observations (Figure 2), but not the deficit of PS genes on the Y chromosome, since the length of human MS Y chromosome genes is in the range of autosomal MS genes (Figure S1B). While we considered a very simple model with only two alleles and associated splicing variants expressed in two tissues, it could readily be extended to accommodate more splicing variants and more than two tissues. However, the main qualitative results concerning the difference between diploid and haploid genes and increased possibility of balancing selection with more tissue specificity are unlikely to change. Note that our model bears some analogies with models explaining the maintenance of genetic diversity in species occupying different ecological niches. Indeed, in those models, selection is spatially heterogeneous, and a stronger habitat preference depending on their genotype leads to an increased chance to globally preserve polymorphism (see e.g. [58]).

### Conclusions

Based on the current evidence and on our theoretical predictions, we can therefore distinguish between the cause and the effect of alternative splicing and propose a model of its evolution. Since favorable conditions for the occurrence of alternative splicing are the existence of splicing variants and their spread and maintenance in the populations, we postulate that alternative splicing is favored and maintained by balancing selection in genes with many exons that are expressed in many tissues. While the width of expression is overall positively correlated with the number of alternative splicing events, this relationship does not seem to explain the lack of genes with alternative splicing variants on the Y chromosome, which is in keeping with a model where balancing selection contributes to the maintenance of alternative splicing. Even though they may not be entirely conserved across evolutionary time [59], the structural characteristics of the genes thus probably determine their splicing status. Indeed, a new splicing variant at a gene expressed in many cellular components would have a larger chance to find a tissue where it would be both advantageous and expressed at a high level than a variant appearing in a gene expressed in a small number of tissues. Our conclusions would certainly benefit from the use of additional data on alternative splicing variants in humans and other species. Complete and accurate information about the extent and conservation of alternative splicing in different species may soon allow one to better understand how this important generator of functional diversity has been shaped across different evolutionary times. Since our model is purely based on cis-regulated altenative splicing, the relative importance of this mode of alternative splicing is an important, yet still unresolved question.

## Supporting Information

Figure S1 A. Distribution of the average longest transcript length (in kb) for MS and PS genes among chromosomes in humans. B. Distribution of the average number of exons for MS and PS genes among chromosomes in humans. In A and B, the range bar correspond to one standard deviation. Uncharacterized genes correspond to ENSEMBL genes that could not be assigned to one of the two other groups, due to insufficient evidence.
(0.28 MB EPS)

Figure S2 A. Total number of splicing events for PS genes in humans and mice as provided by the ASD dataset. B. Number of distinct types of splicing events for PS genes in humans and C. in mice.
(0.43 MB EPS)

Figure S3 Functional classification of MS and PS genes in humans according to the GO ontology. Uncharacterized genes are also shown, for comparison. A gene may be associated with more than one function and hence may belong to more than one functional group. A. Distribution of the number of distinct biological processes. B. Distribution of the number of molecular functions. C. Distribution of the number of cellular components. In all cases, the distributions for MS and PS genes are significantly different (A. Χ^2^ = 74.68, df = 6, p<0.001; B. Χ^2^ = 126.97, df = 3, p<0.001; C. Χ^2^ = 174.36, df = 5, p<0.001), with a clear excess of PS genes in the higher numbers of distinct functional classes.
(0.39 MB EPS)

Figure S4 Functional classification of MS and PS genes in humans according to the GO ontology. Uncharacterized genes are also shown, for comparison. A. Distribution of biological process. B. Distribution of molecular function. C. Distribution of cellular component. The proportion of genes corresponds to the number of genes found in a functional category, scaled to 100% across the whole set of categories. In all cases, we found a significant different distribution of genes among functional groups (A. Χ^2^ = 131.54, df = 7, p<0.001; B. Χ^2^ = 157.34, df = 10, p<0.001; C. Χ^2^ = 252.52, df = 9, p<0.001).
(0.94 MB EPS)

Figure S5 Expression profiles of MS and PS genes in humans using ESTs and the eVOC ontology (see Methods). Uncharacterized genes are also shown, for comparison. A. Distribution of the number of distinct anatomical systems. B. Distribution of the number of cell types. C. Distribution of the number of development stages. D. Distribution of the number of pathologies. In all cases, the distributions for MS and PS genes are different, with PS genes being more widely expressed in space and time (A. Χ^2^ = 2120.39, df = 11, p<0.001; B. Χ^2^ = 1386.81, df = 22, p<0.001; C. Χ^2^ = 1710.46, df = 5, p<0.001, D. Χ^2^ = 1926.54, df = 7, p<0.001).
(0.43 MB EPS)

Figure S6 Expression profiles of MS and PS genes in humans. Uncharacterized genes are also shown, for comparison. A. Distribution of anatomical systems. B. Distribution of cell types. C. Distribution of development stages. D. Distribution of pathologies. The proportion of genes corresponds to the number of genes expressed in a spatial or temporal category, scaled to 100% across the whole set of categories. In all cases, we found a significant different distribution of genes among transcriptional categories (A. Χ^2^ = 561.45, df = 11, p<0.001; B. Χ^2^ = 364.93, df = 30, p<0.001; C. Χ^2^ = 534.12, df = 5, p<0.001; D. Χ^2^ = 691.43, df = 7, p<0.001).
(1.14 MB EPS)

Figure S7 A. Chromosomal distribution of MS and PS genes in mice. ENSEMBL genes that could not be assigned to one of the 2 groups, due to insufficient evidence, are also shown. B. Distribution of the average longest transcript length (in kb) for MS, PS and uncharacterized genes among chromosomes in mice. C. Distribution of the average number of exons for MS, PS and uncharacterized genes among chromosomes in mice. In B. and C., the range displayed corresponds to the mean plus and minus one standard deviation.
(0.39 MB EPS)

Figure S8 Conservation of splicing patterns between humans and mice are displayed here as plots of the numbers of different types of splicing events for pairs of orthologous genes. Pearson's correlation coefficient (r) and associated p-values are shown.
(0.68 MB EPS)

Table S1 List of functional classes, used in this paper, for the three GO ontologies, to describe the function of MS and PS genes (see main text).
(0.10 MB XLS)

Table S2 List of transcriptional classes, used in this paper, to describe the expression profile of MS and PS genes (see main text).
(0.10 MB XLS)

Table S3 Pearson correlation coefficients between the number of functional groups, when comparing the three GO categories, for MS and PS genes. 1. The correlation was computed when considering only the genes annotated in both functional categories. 2. We included the genes that were uncharacterized in one or the two functional categories.
(0.10 MB XLS)

Table S4 Pearson correlation coefficients between the number of expression evidence for MS and PS genes. 1. The correlation was computed when considering only the genes expressed in both transcriptional categories. 2. We included the genes for which we could not detect expression evidence in one or the two transcriptional categories.
(0.10 MB XLS)
